# Supplementary material for: Tissue localization of natural killer cells dictates surveillance of lung metastasis
Source: Nat Commun. 2025 Oct 27;16:9464. doi: 10.1038/s41467-025-64531-7 (PMC12559431; doi:10.1038/s41467-025-64531-7)
Supplement: Supplementary file 1 — Supplementary Information [file 41467_2025_64531_MOESM1_ESM.pdf]

Figure S1

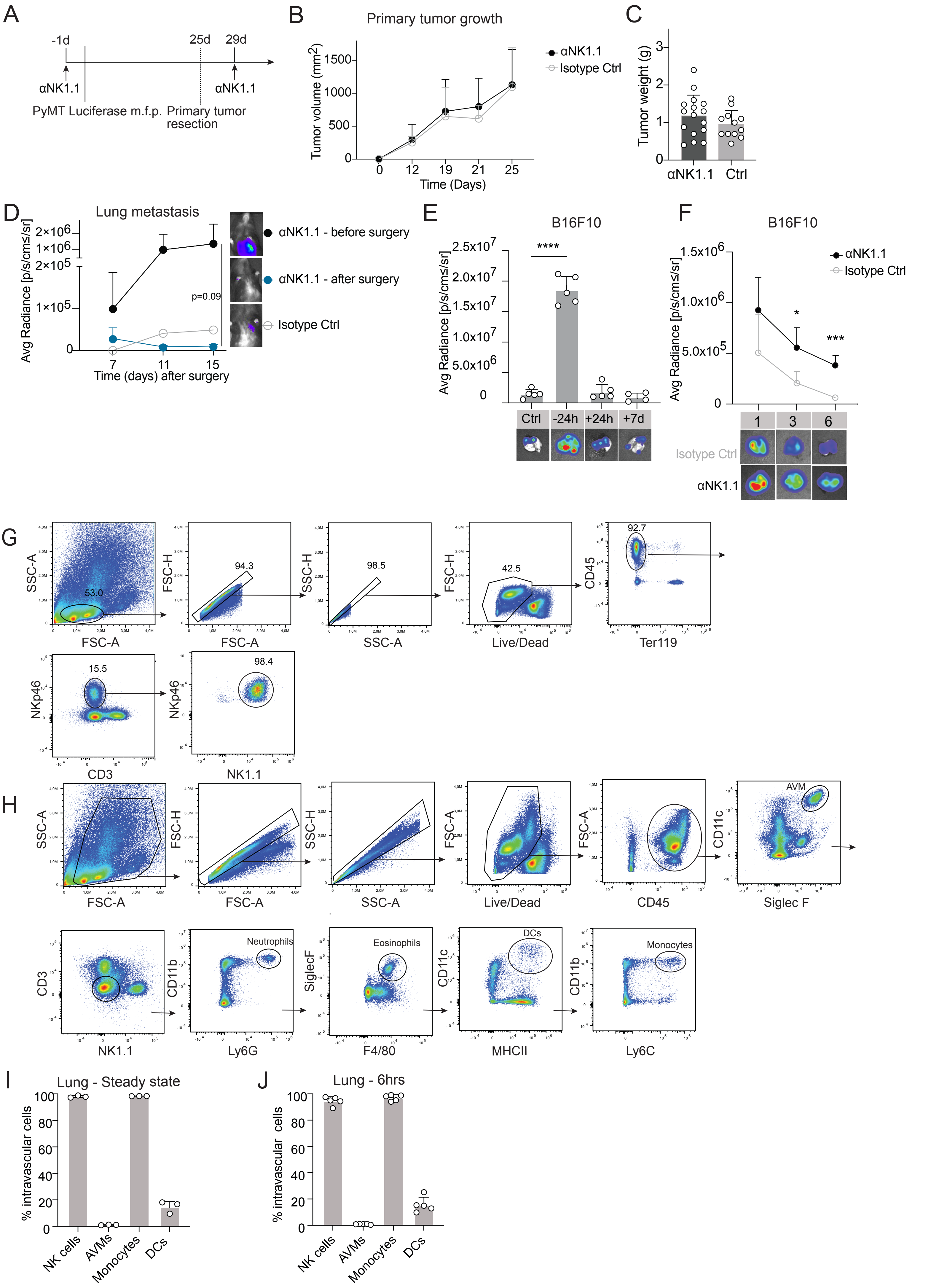

A
-1d
25d
29d
αNK1.1
αNK1.1
PyMT Luciferase m.f.p.
Primary tumor resection
B
Primary tumor growth
Tumor volume (mm2)
Time (Days)
αNK1.1
Isotype Ctrl
C
Tumor weight (g)
αNK1.1
Ctrl
D
Lung metastasis
Avg Radiance [p/s/cm≤/sr]
p=0.09
Time (days) after surgery
αNK1.1 - before surgery
αNK1.1 - after surgery
Isotype Ctrl
E
B16F10
****
Ctrl
-24h
+24h
+7d
F
B16F10
αNK1.1
Isotype Ctrl
*
***
Isotype Ctrl
αNK1.1
G
53.0
94.3
98.5
42.5
92.7
15.5
98.4
SSC-A
FSC-A
FSC-H
SSC-H
Live/Dead
CD45
Ter119
NKp46
CD3
NK1.1
H
AVM
CD11c
Siglec F
CD45
Neutrophils
Eosinophils
DCs
Monocytes
CD11b
Ly6G
SiglecF
F4/80
MHCII
Ly6C
I
Lung - Steady state
% intravascular cells
NK cells
AVMs
Monocytes
DCs
J
Lung - 6hrs

Supplementary figures

Figure S1

(A-D) PyMT Luciferase cells ($5x10^5$) were injected into the fourth mammary fat pad (m.f.p.) to assess metastatic clearance. Primary tumors were resected 25 days after tumor inoculation. NK cells were depleted using 200µg of anti-NK1.1 antibody either 1 day before tumor injection or 4 days after tumor resection. **(A)** Schematic representation of the experimental design. **(B)** Primary tumor growth over time (mm^2) in mice treated with anti-NK1.1 antibody or isotype control. **(C)** Tumor weight (g) at the day of resection in anti.NK1.1- or control-treated mice. **(D)** Kinetics of metastatic load in the lungs measured by IVIS, with representative IVIS images of metastatic lungs from each group at 15 days post-resection. Error bars display means ± SEM. Statistical significance was determined using Two-tailed T test with Welch's correction. **(E)** B16-F10 luciferase cells ($5x10^5$) were injected i.v.. NK cells were depleted using 200ug of αNK1.1 antibody 24h before, 24h after or 7 days after tumor cell inoculation. Tumor cells in the lungs were quantified at day 14 after tumor cell injection using IVIS. Quantification of B16-F10 lung metastasis by in vivo imaging system (IVIS) at the endpoint. Bioluminescent measurements on the whole lungs are shown. **(F)** Early tumor cell clearance in the lungs of mice injected i.v. with B16-F10 Luciferase cells ($5x10^5$) from 1h to 6h after tumor injection. Kinetics of metastatic load in the lungs measured by IVIS with representative IVIS images of metastatic lungs from each group at the different timepoints. **(G)** Gating strategy of NK cells in the lung. NK cells were gated as single live $CD45^+Lineage^-$ ($CD3^-CD5^-CD19^-Ly6G^-Ter119^-$) $NK1.1^+NKp46^+$ cells. **(H)** Gating strategy used for the indicated cell types within the lung. Alveolar macrophages (AVM) were gated as single live $CD45^+CD11c^+SiglecF^+$ cells. Neutrophils were gated as single live $CD45^+$ $CD3^-NK1.1^-$ $CD11b^+Ly6G^+$ cells. Eosinophils were gated single live $CD45^+CD3^-NK1.1^-$ $CD11b^-Ly6G^-$ SiglecF+F4/80+ cells. Dendritic cells (DC) were gated as single live $CD45^+CD3^-NK1.1^-$ $CD11b^-Ly6G^-$ $SiglecF^-CD11c^+MHC\text{-}II^+$ cells and monocytes were gated as single live $CD45^+CD3^-$ $NK1.1^-$ $CD11b^-Ly6G^-SiglecF^-CD11b^+Ly6C^+$ cells. **(I)** Bar graph displaying the percentage of $CD45iv^+$ NK cells, AVMs, monocytes and DCs within naïve lungs. **(J)** Bar graph displaying the frequency of intravascular ($CD45iv^+$) NK cells, AVMs, monocytes and DCs 6 h after tumor inoculation. Data are representative for one of 2 independent experiments with n = 3-5 mice per group. Error bars display means ± SD.

Statistical significance was determined using One-way ANOVA with Tukey's multiple comparisons test (A, E, F) or using Two-way ANOVA with Sidak's post hoc test (B). *P < 0.05, **P < 0.01, ***P < 0.001, and ****P < 0.0001. ns, not significant.

Figure S2

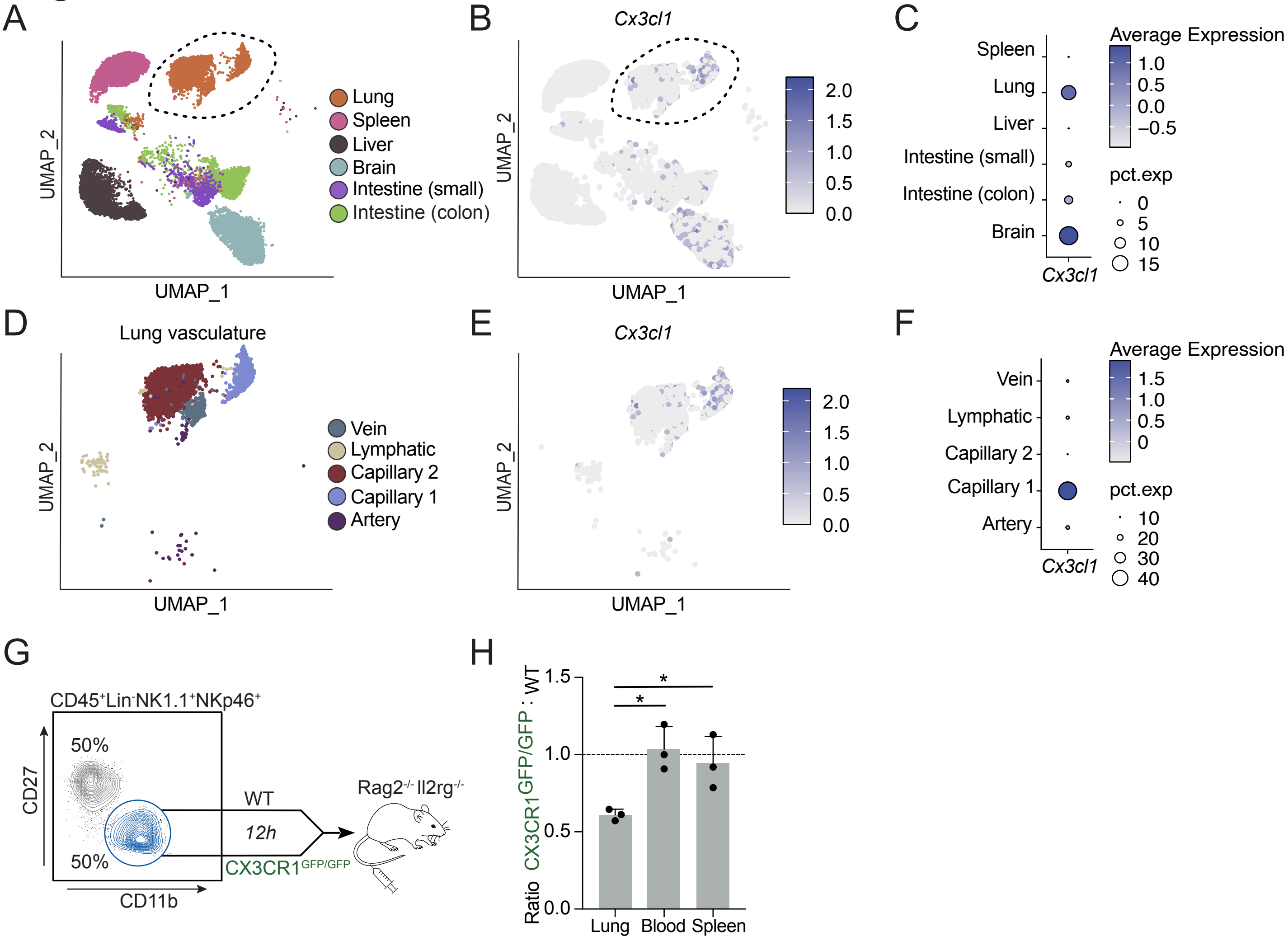

A
UMAP_2
UMAP_1
Lung
Spleen
Liver
Brain
Intestine (small)
Intestine (colon)
B
Cx3cl1
UMAP_2
UMAP_1
2.0
1.5
1.0
0.5
0.0
C
Spleen
Lung
Liver
Intestine (small)
Intestine (colon)
Brain
Cx3cl1
Average Expression
1.0
0.5
0.0
−0.5
pct.exp
0
5
10
15
D
Lung vasculature
UMAP_2
UMAP_1
Vein
Lymphatic
Capillary 2
Capillary 1
Artery
E
Cx3cl1
UMAP_2
UMAP_1
2.0
1.5
1.0
0.5
0.0
F
Vein
Lymphatic
Capillary 2
Capillary 1
Artery
Cx3cl1
Average Expression
1.5
1.0
0.5
0
pct.exp
10
20
30
40
G
CD45+Lin-NK1.1+NKp46+
50%
50%
CD27
CD11b
WT
12h
CX3CR1GFP/GFP
Rag2-/- Il2rg-/-
H
Ratio CX3CR1GFP/GFP : WT
1.5
1.0
0.5
0.0
*
*
Lung
Blood
Spleen

Figure S2

(A-F) Analysis of a scRNA-seq dataset of endothelial cells from different murine organs (Kalucka et al., 2020) (Array express E-MTAB-8077). **(A)** UMAP depicting murine endothelial cell clusters of different organs. **(B)** UMAP displaying CX3CL1 expression on murine endothelium of the different murine organs. **(C)** Dot plot depicting average expression (color intensity) and the percentage of cells expressing *Cx3cl1* (circle size) across each cluster. **(D)** UMAP depicting various pulmonary endothelial cell clusters. **(E)** UMAP displaying *Cx3cl1* expression on pulmonary endothelial clusters. **(F)** Dot plot depicting average expression (color intensity) and the percentage of cells expressing *Cx3cl1* (circle size) across each cluster. **(G-H)** FACS-sorted $CD11b^{high}$ NK cells from wild-type (CD45.1) and $CX3CR1^{GFP/GFP}$ (CD45.2) mice were adoptively transferred at a 1 to 1 ratio in $Rag2^{-/-}Il2rg^{-/-}$ mice and their distribution was analyzed 12h post-transfer. **(G)** Schematic illustration of the experimental workflow. **(H)** Bar chart displaying the ratio of $CX3CR1^{GFP/GFP}$ NK cells versus WT (CD45.1) NK cells across different tissues (lung, blood, and spleen). The ratios are normalized to the corresponding mean blood ratios. Data are representative for one of 2 independent experiments with n = 3-4 mice per group. The bar represents the mean ± SD. Statistical significance was determined using One-way ANOVA with Tukey's multiple comparisons test. *P < 0.05, **P < 0.01, ***P < 0.001, and ****P < 0.0001. ns, not significant.

Figure S3

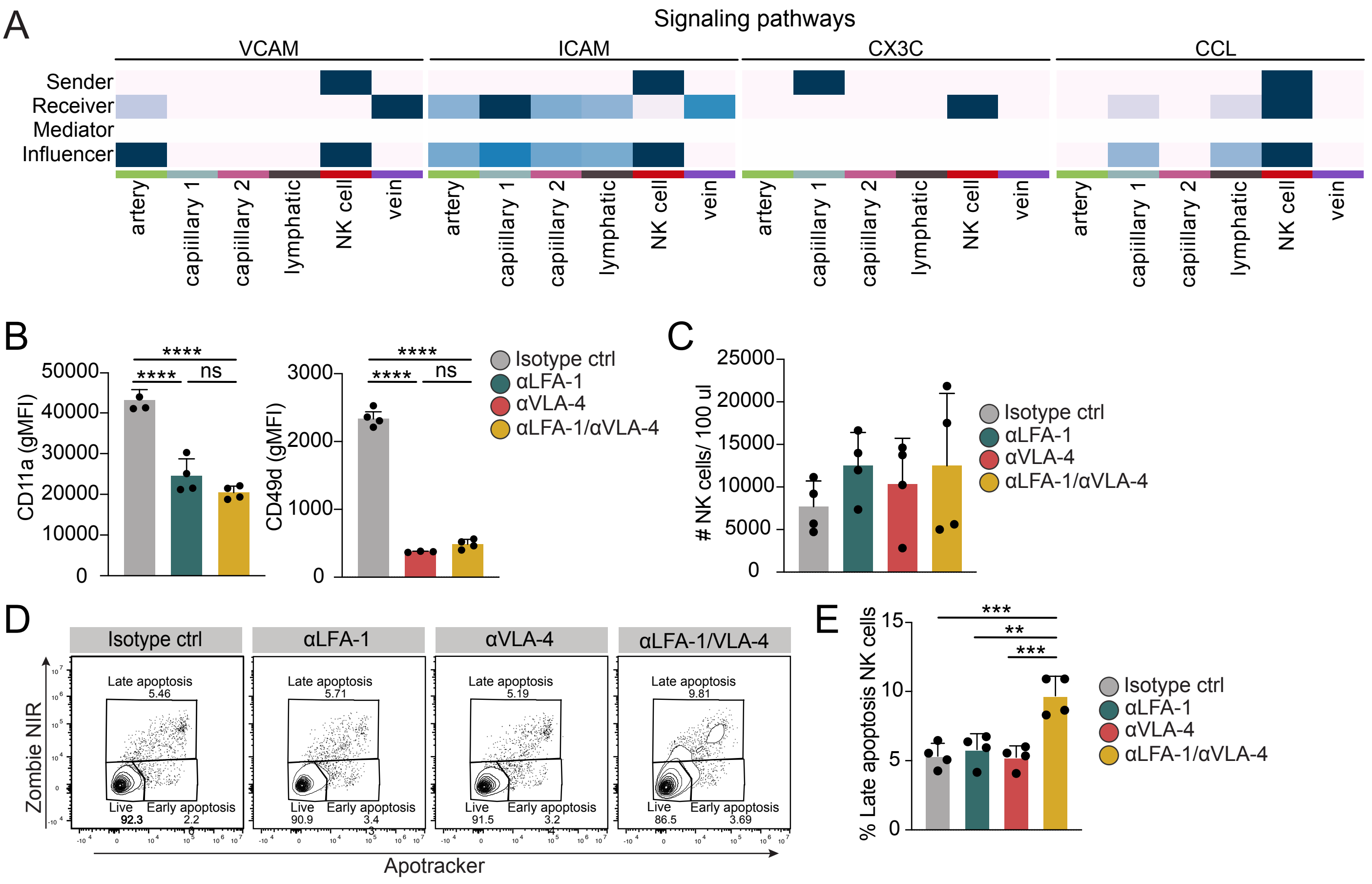

A
Signaling pathways
VCAM
ICAM
CX3C
CCL
Sender
Receiver
Mediator
Influencer
artery
capillary 1
capillary 2
lymphatic
NK cell
vein
Importance
B
CD11a (gMFI)
CD49d (gMFI)
****
ns
Isotype ctrl
αLFA-1
αVLA-4
αLFA-1/αVLA-4
C
NK cells/ 100 ul
D
αLFA-1/VLA-4
Late apoptosis
Live
Early apoptosis
Zombie NIR
Apotracker
E
% Late apoptosis NK cells
***
**

Figure S3

(A) Heatmaps showing the relative importance of each cell group in the VCAM, ICAM, CX3C and CCL signalling pathways based on the computed four network centrality measures by Cellchat. **(B-F)** anti-LFA-1, anti-VLA-4, anti-LFA-1 and anti-VLA-4 or isotype control antibody were intravenously administered and organs were analyzed by flow cytometry 12h after blockade treatment. **(B)** Bar chart showing the gMFI of CD11a (LFA-1) and CD49d (VLA-4). **(C)** Bar chart showing the number of differentiated $CD11b^{high}CD27^{low}$ NK cells in 100 ul of blood. **(D)** Representative contour plots displaying Live ($Apotracker^{-}ZombieNIR^{-}$), early apoptosis ($Apotracker^{+}ZombieNIR^{-}$) and late apoptosis ($Apotracker^{+}ZombieNIR^{+}$) on NK cells. **(E)** Bar chart showing the frequency of NK cells in late apoptosis ($Apotracker^{+}ZombieNIR^{+}$). Data are representative for one of 3 independent experiments with n = 3-5 mice per group. The bar represents the mean ± SD. Statistical significance was determined using One-way ANOVA with Tukey's multiple comparisons test. *P < 0.05, **P < 0.01, ***P < 0.001, and ****P < 0.0001. ns, not significant.

Figure S4

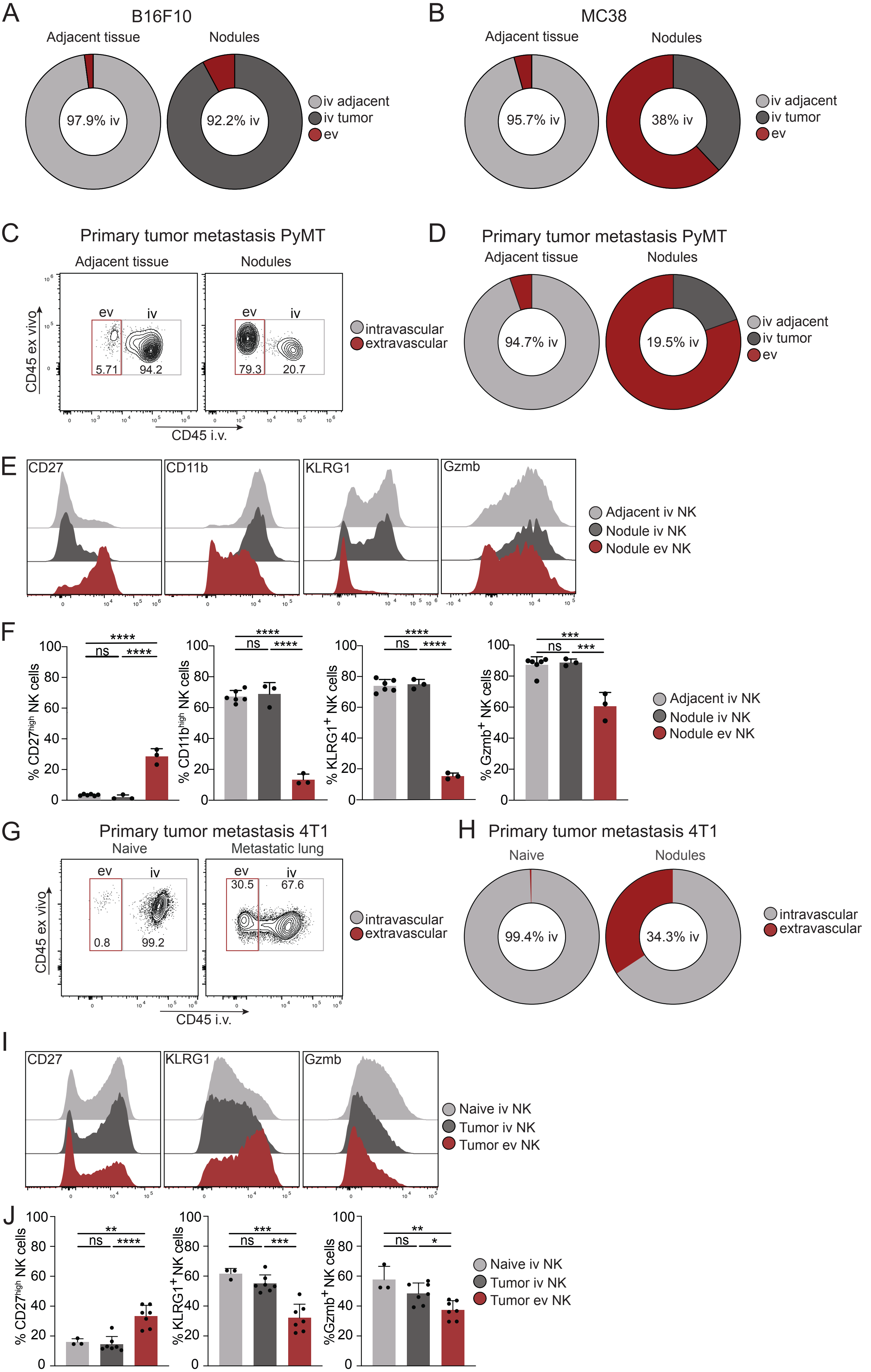
A
B16F10
Adjacent tissue
Nodules
97.9% iv
92.2% iv
iv adjacent
iv tumor
ev
B
MC38
Adjacent tissue
Nodules
95.7% iv
38% iv
iv adjacent
iv tumor
ev
C
Primary tumor metastasis PyMT
Adjacent tissue
Nodules
ev
iv
5.71
94.2
79.3
20.7
CD45 ex vivo
CD45 i.v.
intravascular
extravascular
D
Primary tumor metastasis PyMT
Adjacent tissue
Nodules
94.7% iv
19.5% iv
iv adjacent
iv tumor
ev
E
CD27
CD11b
KLRG1
Gzmb
Adjacent iv NK
Nodule iv NK
Nodule ev NK
F
% CD27high NK cells
% CD11bhigh NK cells
% KLRG1+ NK cells
% Gzmb+ NK cells
ns
****
***
Adjacent iv NK
Nodule iv NK
Nodule ev NK
G
Primary tumor metastasis 4T1
Naive
Metastatic lung
ev
iv
0.8
99.2
30.5
67.6
CD45 ex vivo
CD45 i.v.
intravascular
extravascular
H
Primary tumor metastasis 4T1
Naive
Nodules
99.4% iv
34.3% iv
intravascular
extravascular
I
CD27
KLRG1
Gzmb
Naive iv NK
Tumor iv NK
Tumor ev NK
J
% CD27high NK cells
% KLRG1+ NK cells
%Gzmb+ NK cells
**
ns
****
***
*
Naive iv NK
Tumor iv NK
Tumor ev NK

Figure S4

(A-B) Donut plots showing the mean percentage of intravascular and extravascular NK cells within the adjacent lung tissue (left) and tumor nodules (right) 14 days post injection of **(A)** B16-F10 and **(B)** MC38. **(C-F)** PyMT-Luciferase cells (5×10^5) were injected into the fourth mammary fat pad (m.f.p.). To promote metastatic spread to the lungs, primary tumors were resected 25 days after tumor inoculation. At sacrifice, adjacent lung tissue and tumor nodules were manually separated and analyzed by flow cytometry on day 22 post-resection. **(C)** Representative contour plots showing intravascular and extravascular NK cells in adjacent lung tissue (left) and nodules (right). **(D)** Donut plot showing the mean percentage of adjacent intravascular, adjacent extravascular (left), nodule intravascular and nodule extravascular (right) NK cells. **(E)** Representative histograms depicting expression of CD27, CD11b, KLRG1 and Gzmb in adjacent intravascular, nodule intravascular and nodule extravascular NK cells. **(F)** Bar charts showing the frequency of $CD27^{high}$, $CD11b^{high}$, $KLRG1^{+}$ and $Gzmb^{+}$ in adjacent intravascular, nodule intravascular and nodule extravascular NK cells. **(G-J)** 4T1 cells (5×10^5) were injected into the fourth mammary fat pad (m.f.p.). Mice were sacrificed 28 days post-inoculation. **(G)** Representative contour plots showing intravascular and extravascular NK cells in a naïve lung (left) and metastatic lung (right). **(H)** Donut plot showing the mean percentage of naïve intravascular, adjacent extravascular (left), metastatic intravascular and metastatic extravascular (right) NK cells. **(I)** Representative histograms depicting expression of CD27, KLRG1 and Gzmb in naïve intravascular, nodule intravascular and extravascular NK cells. **(J)** Bar charts showing the frequency of $CD27^{high}$, $KLRG1^{+}$ and $Gzmb^{+}$ in naïve intravascular, nodule intravascular and extravascular NK cells. Error bars display means ± SD. Statistical significance was determined by one way ANOVA with Tukey's multiple comparison test; $^{*}P < 0.05$, $^{**}P < 0.01$, $^{***}P < 0.001$ and $^{****}P < 0.0001$. ns, not significant.

Figure S5

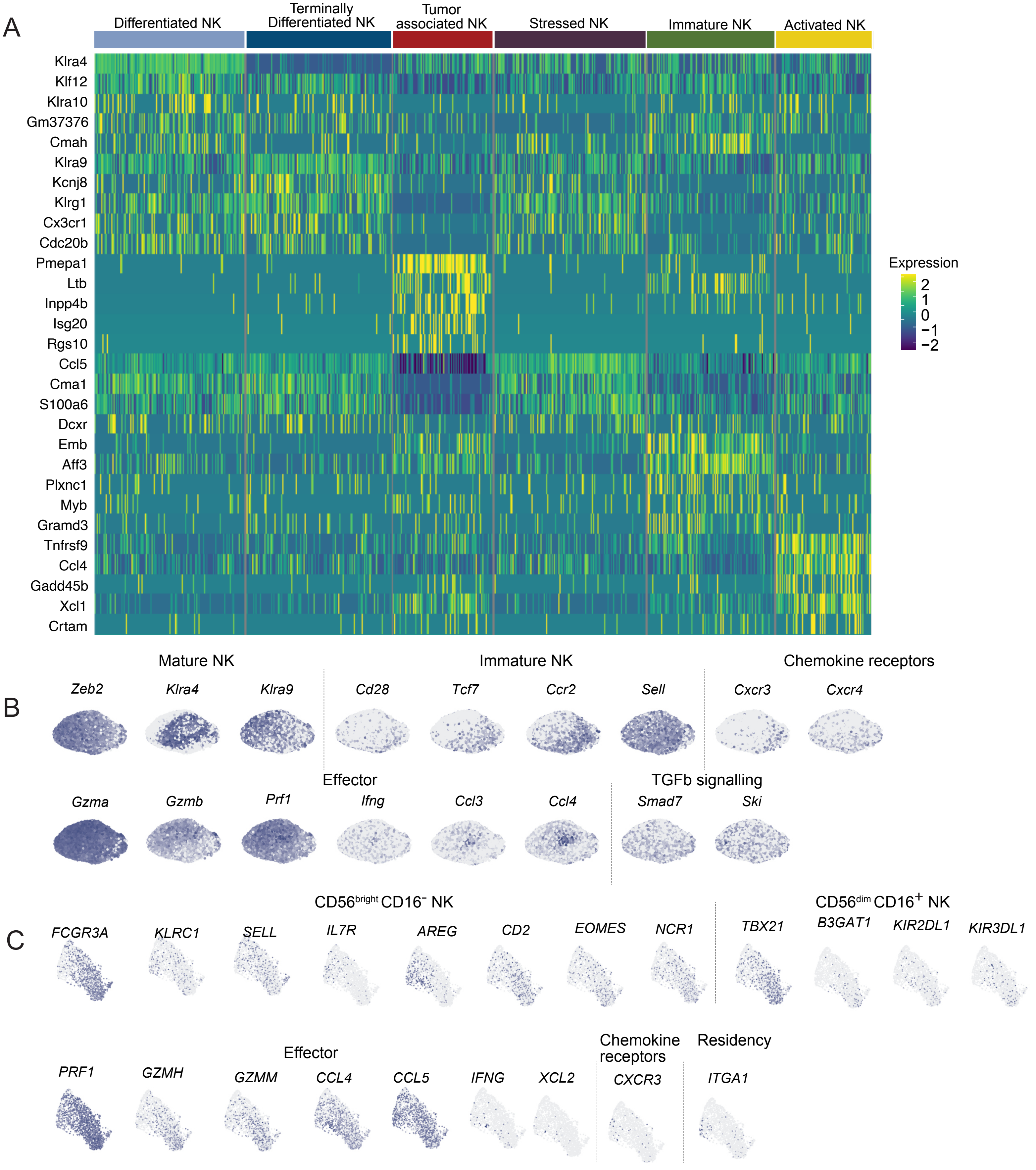
A
Differentiated NK
Terminally Differentiated NK
Tumor associated NK
Stressed NK
Immature NK
Activated NK
Klra4
Klf12
Klra10
Gm37376
Cmah
Klra9
Kcnj8
Klrg1
Cx3cr1
Cdc20b
Pmepa1
Ltb
Inpp4b
Isg20
Rgs10
Ccl5
Cma1
S100a6
Dcxr
Emb
Aff3
Plxnc1
Myb
Gramd3
Tnfrsf9
Ccl4
Gadd45b
Xcl1
Crtam
Expression
2
1
0
−1
−2
B
Mature NK
Zeb2
Klra4
Klra9
Immature NK
Cd28
Tcf7
Ccr2
Sell
Chemokine receptors
Cxcr3
Cxcr4
Effector
Gzma
Gzmb
Prf1
Ifng
Ccl3
Ccl4
TGFb signalling
Smad7
Ski
C
CD56^bright CD16^- NK
FCGR3A
KLRC1
SELL
IL7R
AREG
CD2
EOMES
NCR1
CD56^dim CD16^+ NK
TBX21
B3GAT1
KIR2DL1
KIR3DL1
Effector
PRF1
GZMH
GZMM
CCL4
CCL5
IFNG
XCL2
Chemokine receptors
CXCR3
Residency
ITGA1

Figure S5

(A-B) Transcriptome expression analysis (scRNAseq) of sorted NK cells in the lung and blood was performed on day 14 post-inoculation. **(A)** Heatmap showing average gene expression of most variable markers in the different NK cell clusters. **(B)** UMAP displaying the expression of different genes across the NK cells. **(C)** UMAPS displaying the expression of different genes across the human NK cells of the publicly available scRNA-seq data of (Zilionis et al., 2019).

Figure S6

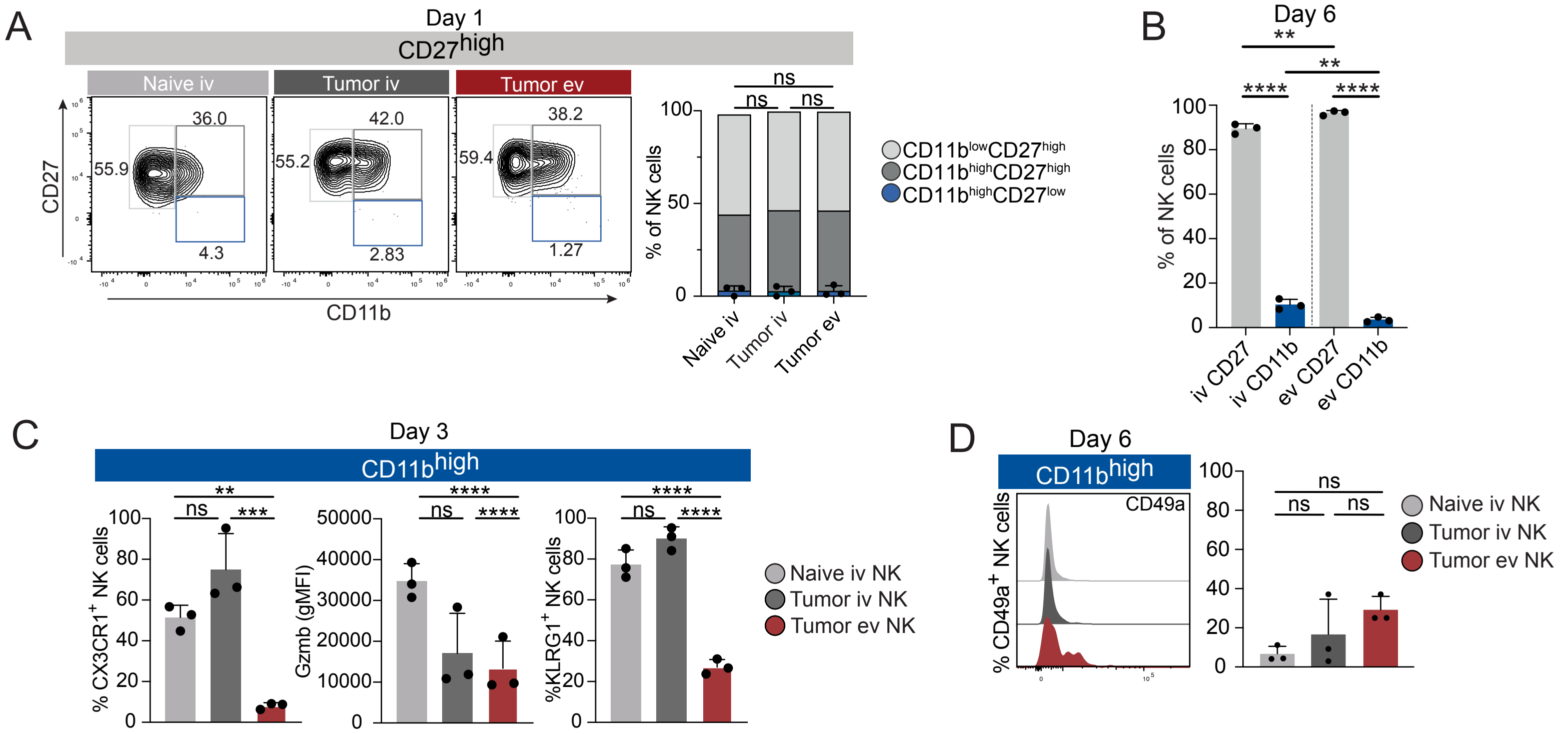

A
Day 1
CD27high
Naive iv
Tumor iv
Tumor ev
36.0
55.9
4.3
42.0
55.2
2.83
38.2
59.4
1.27
CD27
CD11b
% of NK cells
ns
CD11blowCD27high
CD11bhighCD27high
CD11bhighCD27low
Naive iv
Tumor iv
Tumor ev
B
Day 6
**
****
% of NK cells
iv CD27
iv CD11b
ev CD27
ev CD11b
C
Day 3
CD11bhigh
% CX3CR1+ NK cells
Gzmb (gMFI)
%KLRG1+ NK cells
***
Naive iv NK
Tumor iv NK
Tumor ev NK
D
Day 6
CD11bhigh
CD49a
% CD49a+ NK cells

Figure S6

(A) Representative contour plots and bar plot depicting differentiation stages from CD45.1^{+}CD27^{high}-derived NK cells in naïve intravascular, (metastatic) tumor intravascular and tumor extravascular lung tissue at day 6 post transfer. **(B)** Bar charts depicting the frequency of CD45.1^{+}CD27^{high} and CD45.2^{+}CD11b^{high} NK cells in the lung comparing intravascular (iv) and extravascular (ev) NK cells at day 6 post-transfer. **(C)** Bar charts depicting the frequency of CX3CR1^{+}, gMFI of Gzmb, KLRG1^{+} NK cells among CD11b^{high} NK cells in the lung at day 3 post-transfer. All data is representative of 2 (D) or 3 (A-B) different experiments with n= 3-4. Statistical significance was determined by two-way ANOVA with a Sidak's post hoc test (A) or One-way ANOVA with Tukey's multiple comparisons test (B, C). The bar represents the mean ± SD. *P < 0.05, **P < 0.01, ***P < 0.001, and ****P < 0.0001. ns, not significant.

Tables

Table S1

**Antigen**	**Fluorochrome**	**Clone**	**Source**	**Art. Nr**	**Dilution (1:X)**	**Notes**
CD11a	APC	2D7	BioLegend	101120	400	
CD11a	PE	2D7	BD	553121	400	
CD11b	BV605	M1/70	BioLegend	101257	600	
CD11b	BUV737	M1/70	BD	612800	600	
CD11c	PE-Cy5.5	N418	eBioscience	35-0114-82	800	
CD16/32	Purified	93	BioLegend	101310	600	FcReceptor blocking
CD19	Biotin	6D5	BioLegend	115504	400	For Lineage
CD19	BUV661	1D3	BD	612971	600	
CD27	PE-Cy7	LG.3A10	BioLegend	124216	400	
CD29	PE	HMb1.1	eBioscience	12029182	200	
CD3ε	Biotin	145-2C11	BioLegend	100304	200	For Lineage
CD45	BUV395	30-F11	BD	564279	300	
CD45	Pacific Blue	30-F11	Biolegend	103126	400	For i.v. injection, 5 ug
CD45.1	PE	A20	BD	553776	400	
CD45.2	APC-cy7	104	Biolegend	109824	400	
CD45.2	Pacific Blue	104	Biolegend	109820	400	For i.v. injection, 5 ug
CD49a	BV510	Ha31/8	BD	740144	100	
CD49b	FITC	DX5	Biolegend	108906	200	
CD49b	Pacific Blue	DX5	Biolegend	108918	200	
CD49d	BUV805	R1-2	BD	741925	400	
CD5	Biotin	53-7.2	BD	553019	800	For Lineage
CX3CR1	BV785	SA011F11	Biolegend	149029	200	

CXCR3	BV650	CXCR3-73	BioLegend	126531	200	
Eomes	PE-eFluor610	Dan11mag	eBioscience	61-4875-82	300	
F4/80	Biotin	BM8	Biolgened	123105	400	For Lineage
F4/80	BV510	BM8	Biolegend	123135	400	
Granzyme B	AlexaFluor 647	GB11	Biolegend	515406	400	
I-A /I-E	BB700	M5/114.15.2	BD	746197	400	
KLRG1	PE-Dazzle594	KLRG1	Biolegend	138423	400	
Live dead BLUE	/	/	Invitrogen		600	Live dead staining of leukocytes
Live dead Near-IR	/	/	Invitrogen		600	Live dead staining of leukocytes
Ly6C	BV711	HK1.4	Biolegend	128037	400	
Ly6G	Biotin	1A8	Biolegend	127604	400	For Lineage
Ly6G	BV650	1A8	Biolegend	127641	400	
NK1.1	APC	PK136	BD	550627	200	
NK1.1	BB700	PK136	BD	566502	200	
NK1.1	BV711	PK136	BD	108749	200	
NK1.1	BV785	PK136	BD	108706	200	
NK1.1	FITC	PK136	BD	108706	200	
NKp46	FITC	29A1.4	Biolegend	137611	100	
NKp46	PerCPeFluor 710	29A1.4	Biolegend	137606	100	
SiglecF	BV750	E50-2440	BD	747316	400	
Streptavidin	BUV563	/	BD	612935	400	For Lineage
Streptavidin	BUV805	/	BD	565923	400	For Lineage
TCRb	Biotin	H57-597	Biolgened	109203	400	For Lineage
TCRb	APC-cy7	H57-597	Biolegend	109220	400	
Ter119	Biotin	Ter-119	Biolegend	116204	400	For Lineage

Ter119	APC	Ter-119	Invitrogen	17-5921-81	400	

Table S2

**Antigen**	**Host**	**Source**	**Art. Nr.**	**Dilution (1:X)**	**Notes**
CD144	Rat	Biolegend	138019	/	Conjugated to AlexaFluor 647, injected 100 µg i.v.
Alexa Fluor™ 647 NHS-Ester	/	Invitrogen	10769744	/	Conjugated to CD144
VCAM-1	Goat	R&D Systems	AF643	200	
ICAM-1	Goat	R&D Systems	AF796	200	
Anti-goat AlexaFluor 488	Donkey	ThermoFisher	A11055	500	
