# Supplementary material for: Tissue localization of natural killer cells dictates surveillance of lung metastasis
Source: Nat Commun. 2025 Oct 27;16:9464. doi: 10.1038/s41467-025-64531-7 (PMC12559431; doi:10.1038/s41467-025-64531-7)
Supplement: Supplementary file 2 — Reporting Summary [file 41467_2025_64531_MOESM2_ESM.pdf]

nature portfolio

Corresponding author(s): Sonia Tugues

Last updated by author(s): July 16, 2025

Reporting Summary

Nature Portfolio wishes to improve the reproducibility of the work that we publish. This form provides structure for consistency and transparency in reporting. For further information on Nature Portfolio policies, see our Editorial Policies and the Editorial Policy Checklist.

Statistics

For all statistical analyses, confirm that the following items are present in the figure legend, table legend, main text, or Methods section.

n/a	Confirmed	
☐	☒	The exact sample size (*n*) for each experimental group/condition, given as a discrete number and unit of measurement
☐	☒	A statement on whether measurements were taken from distinct samples or whether the same sample was measured repeatedly
☐	☒	The statistical test(s) used AND whether they are one- or two-sided<br>*Only common tests should be described solely by name; describe more complex techniques in the Methods section.*
☐	☒	A description of all covariates tested
☐	☒	A description of any assumptions or corrections, such as tests of normality and adjustment for multiple comparisons
☐	☒	A full description of the statistical parameters including central tendency (e.g. means) or other basic estimates (e.g. regression coefficient) AND variation (e.g. standard deviation) or associated estimates of uncertainty (e.g. confidence intervals)
☐	☒	For null hypothesis testing, the test statistic (e.g. *F*, *t*, *r*) with confidence intervals, effect sizes, degrees of freedom and *P* value noted<br>*Give P values as exact values whenever suitable.*
☒	☐	For Bayesian analysis, information on the choice of priors and Markov chain Monte Carlo settings
☒	☐	For hierarchical and complex designs, identification of the appropriate level for tests and full reporting of outcomes
☒	☐	Estimates of effect sizes (e.g. Cohen's *d*, Pearson's *r*), indicating how they were calculated

Our web collection on statistics for biologists contains articles on many of the points above.

Software and code

Policy information about availability of computer code

Data collection	Flow cytometry data: Cytek Aurora 5; Spectroflo Software (v3.0 & v3.1). BD FACSAria 3 or FACSymphony S6, FACS Diva software v9.1.<br>Immunofluorescence: Leica SP8 Falcon confocal microscope<br>Single cell RNA sequencing: mouse cells were sorted using BD FACS Symphony S6 and loaded into 10x Genomics Chronium. Libraries were prepared according to the manufacturer's instructions and sequenced on an Illumina Novaseq S1.<br>In vivo imaging: IVIS200imaging system (PerkinElmer)
Data analysis	Flow Cytometry data:<br>Flowjo Software (V10, BD)<br>Prism software (Graphpad v9)<br><br>Single Cell RNA Sequencing:<br>R (v5.0), Seurat (v5.0), FastMNN was used for data integration. CellChat was used to predict cell interactions.<br><br>In vivo imaging: Living image (v4.7.1)

For manuscripts utilizing custom algorithms or software that are central to the research but not yet described in published literature, software must be made available to editors and reviewers. We strongly encourage code deposition in a community repository (e.g. GitHub). See the Nature Portfolio guidelines for submitting code & software for further information.

Data

Policy information about availability of data

All manuscripts must include a data availability statement. This statement should provide the following information, where applicable:

- Accession codes, unique identifiers, or web links for publicly available datasets
- A description of any restrictions on data availability
- For clinical datasets or third party data, please ensure that the statement adheres to our policy

Singlecell sequencing data generated for this study have been deposited in the Gene Expression Omnibus (GEO) under accession number GSE301222. The accessibility of publicly available datasets used in this study is as follows: Murine endothelial cells (Kalucka et al., 2020) is available under the accession number E-MTAB-8077 (ArrayExpress), tumor-associated and circulating Natural Killer cells from Non-small cell lung cancer patients (Zilionis et al., 2019) is available under the accession number GSE127465.

Research involving human participants, their data, or biological material

Policy information about studies with human participants or human data. See also policy information about sex, gender (identity/presentation), and sexual orientation and race, ethnicity and racism.

Reporting on sex and gender	NA
Reporting on race, ethnicity, or other socially relevant groupings	NA
Population characteristics	NA
Recruitment	NA
Ethics oversight	NA

Note that full information on the approval of the study protocol must also be provided in the manuscript.

Field-specific reporting

Please select the one below that is the best fit for your research. If you are not sure, read the appropriate sections before making your selection.

☒ Life sciences ☐ Behavioural & social sciences ☐ Ecological, evolutionary & environmental sciences

For a reference copy of the document with all sections, see nature.com/documents/nr-reporting-summary-flat.pdf

Life sciences study design

All studies must disclose on these points even when the disclosure is negative.

Sample size	We did not perform any sample size calculations. The sample size in flow cytometry experiments was greater than or equal to 3 mice per group, 2-3 independent experiments are performed. For single cell RNA sequencing 5 biological replicates were chosen, since only one independent experiment was performed.
Data exclusions	No animals were excluded from the analysis.
Replication	Experiments were succesfully repeated and the number of experiments is stated in the figure legends.<br><br>scRNA sequencing was performed once, since sequencing results are highly robust due to the high number of individual cells. In addition, scRNAseq experiments are cost-intensive and therefore it is common practice to only perform them once.
Randomization	Mice were grouped by genotype (when using NKp46-cre/Tgfbr2fl and control mice).
Blinding	Tumor inoculations, treatments and measurements of primary tumor size by caliper gauge were performed in a blinded fashion.

Reporting for specific materials, systems and methods

We require information from authors about some types of materials, experimental systems and methods used in many studies. Here, indicate whether each material, system or method listed is relevant to your study. If you are not sure if a list item applies to your research, read the appropriate section before selecting a response.

Materials & experimental systems

n/a	Involved in the study
☐	☒ Antibodies
☐	☒ Eukaryotic cell lines
☒	☐ Palaeontology and archaeology
☐	☒ Animals and other organisms
☒	☐ Clinical data
☒	☐ Dual use research of concern
☒	☐ Plants

Methods

n/a	Involved in the study
☒	☐ ChIP-seq
☐	☒ Flow cytometry
☒	☐ MRI-based neuroimaging

Antibodies

Antibodies used

Anti-mouse antibodies including anti-CD11a (APC, clone 2D7, 1:400 dilution), anti-CD11b (BV605, clone M1/70, 1:600 dilution), anti-CD11c (PE-Cy5.5, clone N418, 1:800 dilution), anti-CD16/32 (Purified, clone 93, 1:600 dilution), anti-CD19 (Biotin, clone 6D5, 1:400 dilution), anti-CD27 (PE-Cy7, clone LG.3A10, 1:400 dilution), anti-CD3ε (Biotin, clone 145-2C11, 1:200 dilution), anti-CD45 (Pacific Blue, clone 30-F11, 1:400 dilution), anti-CD45.2 (APC-Cy7, clone 104, 1:400 dilution), anti-CD45.2 (Pacific Blue, clone 104, 1:400 dilution), anti-CD49b (FITC, clone DX5, 1:200 dilution), anti-CD49b (Pacific Blue, clone DX5, 1:200 dilution), anti-CX3CR1 (BV785, clone SA011F11, 1:200 dilution), anti-CXCR3 (BV650, clone CXCR3-73, 1:200 dilution), anti-F4/80 (Biotin, clone BM8, 1:400 dilution), anti-F4/80 (BV510, clone BM8, 1:400 dilution), anti-Granzyme B (Alexa Fluor 647, clone GB11, 1:400 dilution), anti-KLRG1 (PE-Dazzle 594, clone KLRG1, 1:400 dilution), anti-Ly6C (BV711, clone HK1.4, 1:400 dilution), anti-Ly6G (Biotin, clone 1A8, 1:400 dilution), anti-Ly6G (BV650, clone 1A8, 1:400 dilution), anti-NKp46 (FITC, clone 29A1.4, 1:100 dilution), anti-NKp46 (PerCP-eFluor 710, clone 29A1.4, 1:100 dilution), anti-TCRβ (Biotin, clone H57-597, 1:400 dilution), anti-TCRβ (APC-Cy7, clone H57-597, 1:400 dilution), and anti-Ter119 (Biotin, clone Ter-119, 1:400 dilution) were obtained from BioLegend.

Anti-mouse antibodies including anti-CD11a (PE, clone 2D7, 1:400 dilution), anti-CD19 (BUV661, clone 1D3, 1:600 dilution), anti-CD45 (BUV395, clone 30-F11, 1:300 dilution), anti-CD45.1 (PE, clone A20, 1:400 dilution), anti-CD49a (BV510, clone Ha31/8, 1:100 dilution), anti-CD49d (BUV805, clone R1-2, 1:400 dilution), anti-CD5 (Biotin, clone 53-7.2, 1:800 dilution), anti-Ly6G (BV650, clone 1A8, 1:400 dilution), anti-NK1.1 (APC, clone PK136, 1:200 dilution), anti-NK1.1 (BB700, clone PK136, 1:200 dilution), anti-NK1.1 (BV711, clone PK136, 1:200 dilution), anti-NK1.1 (BV785, clone PK136, 1:200 dilution), anti-NK1.1 (FITC, clone PK136, 1:200 dilution), anti-SiglecF (BV750, clone E50-2440, 1:400 dilution), Streptavidin (BUV563, 1:400 dilution), and Streptavidin (BUV805, 1:400 dilution) were obtained from BD Biosciences.

Anti-mouse antibodies including anti-CD11c (PE-Cy5.5, clone N418, 1:800 dilution), anti-CD29 (PE, clone HMb1.1, 1:200 dilution), and anti-Eomes (PE-eFluor 610, clone Dan11mag, 1:300 dilution) were obtained from eBioscience.

Anti-mouse antibodies including anti-MHC class II (I-A/I-E) (BB700, clone M5/114.15.2, 1:400 dilution) were obtained from BD.

Anti-mouse antibodies including anti-Ter119 (APC, clone Ter-119, 1:400 dilution) were obtained from Invitrogen.

Live/Dead staining reagents including Live/Dead Blue (Invitrogen, 1:600 dilution) and Live/Dead Near-IR (Invitrogen, 1:600 dilution) were used to exclude dead leukocytes.

Validation

All antibodies in our study are commercially available and have been titrated in-house. All antibodies have been validated by the commercial manufacturers. Validation data are available on the manufacturer's website.

Eukaryotic cell lines

Policy information about cell lines and Sex and Gender in Research

Cell line source(s)

PyMT cells were a gift from David G DeNardo (Division of Oncology, Washington University School of Medicine). MC38 and B16-F10 melanoma cells were purchased from ATCC. 4T1 cells were provided by Prof. Michael Detmar.

Authentication

None of the cell lines were authenticated in these studies, for experiments cell lines with low passage numbers were used.

Mycoplasma contamination

All cell lines used in the study tested negative for mycoplasm contamination.

Commonly misidentified lines (See ICLAC register)

No commonly misidentified lines were used.

Animals and other research organisms

Policy information about studies involving animals; ARRIVE guidelines recommended for reporting animal research, and Sex and Gender in Research

Laboratory animals

6- to 10-week-old female and male C57BL/6 mice were purchased from Janvier Labs. Ncr1Cre/wt mice (B6.Ncr1tm1.1(icre)Viv) were provided by Eric Vivier. Ai14fl/fl (B6;129S6-Gt(ROSA)26Sortm14(CAG-tdTomato)Hze/J, stock# 007908), C57BL/6-LY5.1 (CD45.1) mice, Tgfbr2fl (B6;129-Tgfbr2tm1Karl/J, stock# 012603), Tgfb1fl (C57BL/6J-Tgfb1em2Lutzy/Mmjax, stock# 065809), Rag2−/−Il2ry−/− (C;129S4-Rag2tm1.1Flvll2rgtm1.1Flv/J, stock #014593), CX3CR-1GFP knock-in/knock-out (B6.129P2(Cg)-Cx3cr1tm1Litt/J, stock#

	005582) were purchased from the Jackson Laboratory. All mice were maintained on a C57BL/6 background and were housed in a pathogen-free environment. Mice were used for experiments at the age of 6-10 weeks. Mice were socially housed with a dark-light cycle of 12h and 45-65% humidity under specific-pathogen-free conditions according to institutional guidelines in the Laboratory Animal Services Center of the University of Zurich.
Wild animals	This study did not involve wild animals.
Reporting on sex	Findings did not apply to one sex.
Field-collected samples	This study did not involve field-collected samples.
Ethics oversight	All experiments were approved by the Cantonal Veterinary Office of Zurich.

Note that full information on the approval of the study protocol must also be provided in the manuscript.

Plants

Seed stocks	NA
Novel plant genotypes	NA
Authentication	NA

Flow Cytometry

Plots

Confirm that:

☐ The axis labels state the marker and fluorochrome used (e.g. CD4-FITC).

☒ The axis scales are clearly visible. Include numbers along axes only for bottom left plot of group (a 'group' is an analysis of identical markers).

☒ All plots are contour plots with outliers or pseudocolor plots.

☒ A numerical value for number of cells or percentage (with statistics) is provided.

Methodology

Sample preparation	Lungs were minced into pieces using the gentleMACS dissociator (program m_lung_01_02, Miltenyi Biotec) in digestion buffer (HBSS) with calcium/magnesium supplemented with 2% FCS10 mM HEPES, 30 μg ml–1 DNase I and 0.4mg/ml collagenase IV. Similarly, spleens were cut manually, and the organs were digested in digestion buffer for 30 min at 37 °C with gentle rocking. For the lung, this was followed by further dissociation on the gentleMACS Dissociator (program m_lung_02_01). Organs were filtered through 70-μm cell strainers and washed and incubated in ACK (ammonium-chloride-potassium, Sigma-Aldrich) buffer for 3 min to lyse erythrocytes.<br>Blood was collected prior to perfusion from the heart and collected in Li-heparin–coated tubes (Sarstedt) and incubated for 15 min in ACK buffer at 4°C with gentle rocking.
Instrument	Cells were analyzed using a Cytek Aurora 5L spectral flow cytometer, cells were sorted usingBD FACSAria 3 or FACSymphony S6
Software	Spectroflow (Cytek) or BD FACS Diva was used for data acquisition, Flowjo (BD) v10 was used for data analysis.
Cell population abundance	For single cell RNA sequencing Live NK cells after FACS purification had a purity of >99%.
Gating strategy	FSC-A and SSC-a gating was applied to exclude debris, doublets were excluded by FSC-Area vs. FSC-Height gating. Dead cells were excluded using Live/Dead Blue (Invitrogen) and Live/Dead Near-IR (Invitrogen). Gating strategies for relevant cells included:<br>NK cells: CD45+CD3-CD5-CD19-Ly6G-Ter119- NK1.1+NKp46+<br>Alveolar macrophages: CD45+CD11c+SiglecF+<br>Neutrophils: CD45+CD3-NK1.1- CD11b+Ly6G+<br>Eosinophils: CD45+CD3-NK1.1- CD11b-Ly6G- SiglecF+F4/80+<br>Dendritic cells: CD45+CD3-NK1.1- CD11b-Ly6G-SiglecF-CD11c+MHC-II+<br>Monocytes: CD45+CD3-NK1.1- CD11b-Ly6G-SiglecF-CD11b+Ly6C+

☒ Tick this box to confirm that a figure exemplifying the gating strategy is provided in the Supplementary Information.
